# Personalized Prediction of Response to Smartphone-Delivered Meditation Training: Randomized Controlled Trial

**DOI:** 10.2196/41566

**Published:** 2022-11-08

**Authors:** Christian A Webb, Matthew J Hirshberg, Richard J Davidson, Simon B Goldberg

**Affiliations:** 1 Harvard Medical School Boston, MA United States; 2 McLean Hospital Belmont, MA United States; 3 Center for Healthy Minds University of Wisconsin – Madison Madison, WI United States; 4 Department of Psychology University of Wisconsin – Madison Madison, WI United States; 5 Department of Psychiatry University of Wisconsin – Madison Madison, WI United States; 6 Department of Counseling Psychology University of Wisconsin – Madison Madison, WI United States

**Keywords:** precision medicine, prediction, machine learning, meditation, mobile technology, smartphone app, mobile phone

## Abstract

**Background:**

Meditation apps have surged in popularity in recent years, with an increasing number of individuals turning to these apps to cope with stress, including during the COVID-19 pandemic. Meditation apps are the most commonly used mental health apps for depression and anxiety. However, little is known about who is well suited to these apps.

**Objective:**

This study aimed to develop and test a data-driven algorithm to predict which individuals are most likely to benefit from app-based meditation training.

**Methods:**

Using randomized controlled trial data comparing a 4-week meditation app (Healthy Minds Program [HMP]) with an assessment-only control condition in school system employees (n=662), we developed an algorithm to predict who is most likely to benefit from HMP. Baseline clinical and demographic characteristics were submitted to a machine learning model to develop a “Personalized Advantage Index” (PAI) reflecting an individual’s expected reduction in distress (primary outcome) from HMP versus control.

**Results:**

A significant group × PAI interaction emerged (*t*_658_=3.30; *P*=.001), indicating that PAI scores moderated group differences in outcomes. A regression model that included repetitive negative thinking as the sole baseline predictor performed comparably well. Finally, we demonstrate the translation of a predictive model into personalized recommendations of expected benefit.

**Conclusions:**

Overall, the results revealed the potential of a data-driven algorithm to inform which individuals are most likely to benefit from a meditation app. Such an algorithm could be used to objectively communicate expected benefits to individuals, allowing them to make more informed decisions about whether a meditation app is appropriate for them.

**Trial Registration:**

ClinicalTrials.gov NCT04426318; https://clinicaltrials.gov/ct2/show/NCT04426318

## Introduction

### Background

Precision medicine, which involves the use of individual variability to guide prevention and treatment, has gained momentum in the health sciences over the past several years [1]. This approach aims to improve outcomes by matching patients with interventions most likely to yield success. In some medical specialties, precision medicine has led to impressive advances in personalized care. For example, research in oncology (eg, lung and breast cancer) has effectively matched patients to targeted cancer treatments based on the unique genetic characteristics of their tumors, which has been shown to improve outcomes [2-4].

Psychiatry and clinical psychology have long hoped to better match patients with interventions. Numerous studies have examined patient-level factors (eg, demographic, clinical, and neurobiological characteristics) as predictors of treatment response [5,6]. However, with many potential predictors and inconsistencies across studies in the presence, direction, and strength of associations with outcomes, empirically supported guidelines for optimal treatment matching remain elusive.

Machine learning has emerged as a promising analytical approach well suited for handling and integrating large numbers of predictor variables, including correlated predictors, that may individually only modestly predict outcomes of interest but can collectively predict significant variance in patient outcomes [7,8]. Specific machine learning approaches such as decision tree–based algorithms (eg, random forest) also effectively model nonlinear and higher-order interactions that may underlie predictive relationships [9]. In contrast to traditional statistical approaches that emphasize evaluating a specific hypothesis (ie, null hypothesis significance testing), machine learning models typically emphasize optimizing predictive performance, and evaluating the generalizability of models to new individuals (eg, via cross-validation [CV], hold-out samples, or external validation) [10]. Machine learning approaches are increasingly being applied with some success in psychiatry and clinical psychology, with a growing number of studies demonstrating their ability to predict response to various psychiatric treatments [10-12].

In pursuit of precision mental health, researchers have leveraged machine learning approaches to optimize treatment recommendations [13-15]. For example, DeRubeis et al [16] developed the *Personalized Advantage Index* (PAI) as an algorithm for guiding treatment recommendations based on pretreatment patient characteristics. These models attempt to predict the benefit that a specific patient would derive from treatment A versus treatment B. The PAI has been successfully used to predict response to cognitive behavioral therapy (CBT) versus an antidepressant medication [16], CBT versus interpersonal therapy [17], CBT versus psychodynamic therapy [18], and an antidepressant medication versus placebo [19].

Prior research using the PAI and related approaches [12] provides promising initial evidence that data-driven algorithms may improve patient outcomes by matching individuals to the most therapeutically beneficial treatment, as opposed to the current suboptimal trial and error approach to treatment selection, which results in protracted psychiatric illness until an effective treatment is found. However, the fact remains that a substantial proportion of individuals with psychiatric disorders go untreated [20,21]. Digital health technologies, such as internet-based CBT [22] and smartphone-delivered mental health apps [23], have the potential to substantially increase access to evidence-based treatments [24]. However, the availability of thousands of mental health apps leaves potential consumers faced with a dizzying number of choices, with essentially no way of knowing which specific app may best suit their needs [25]. Data-driven treatment recommendation algorithms, such as the PAI, offer promising tools for informing optimal patient-treatment fit. Such approaches may also be valuable for addressing the persistent limitations of mobile health (mHealth) approaches, including notoriously high and rapid disengagement [26,27]. Moreover, the scalability of mHealth makes it possible to collect adequately powered sample sizes for robust modeling [28].

A recent analysis of available mental health apps revealed that meditation and mindfulness training (along with journaling and mood tracking) are the most common features offered across apps [29]. The two most widely used meditation apps (Headspace and Calm, with 5 million and 9 million monthly active users, respectively) account for 96% of daily active users in a recent evaluation of the top 27 apps for depression and anxiety [30]. Despite the soaring popularity of meditation apps, a critical question remains unanswered: *For whom* is app-based meditation training well suited?

### This Study

This study involved a secondary analysis of a large-scale randomized controlled trial (RCT) comparing a meditation-based smartphone app, the Healthy Minds Program (HMP), with an assessment-only control condition [31]. The RCT was conducted on a sample of school district employees (n=662) in the state of Wisconsin during the COVID-19 pandemic. Relative to prepandemic levels, the rates of emotional distress and depressive symptoms increased substantially during the COVID-19 pandemic [32]. Available evidence suggests that the emotional well-being of teachers also decreased during the pandemic [33,34], as they coped with COVID-19–related stressors, uncertainty, and risks with the return to in-person instruction. Using data from the above RCT, the overarching goal of this study was to develop and evaluate a data-driven (PAI) approach to inform personalized meditation app recommendations for school employees. Using readily gathered self-reported baseline demographic and clinical characteristics, we developed and tested a machine learning algorithm to identify which individuals are most likely to benefit from the HMP app.

## Methods

### Participants and Procedure

Wisconsin school district employees were recruited via email and other electronic media between mid-June 2020 and late August 2020 (for a full description of study procedures, refer to the study by Hirshberg et al [31]). Eligible participants were adults (aged ≥18 years) currently employed by a Wisconsin school who owned a smartphone capable of downloading the HMP, were fluent in English, had limited exposure to meditation or the HMP app, and had depressive symptoms below the severe range (*t* score<70 on Patient-Reported Outcomes Information System [PROMIS] Depression [35]). The *t* scores are population normed at 50, with an SD of 10. On completing the pretest measures, 666 participants were randomly assigned to use the 4-week HMP or an assessment-only control condition (4 participants were removed for failing multiple attention checks; refer to Figure S2 in Multimedia Appendix 1 [31,35-49] for the CONSORT [Consolidated Standards of Reporting Trials] flow diagram). Participants completed weekly questionnaires during the intervention period (ie, weeks 1, 2, and 3) along with a posttreatment assessment (week 4) and follow-up assessment (3 months after the end of the intervention period). These measures were administered via the web-based REDCap (Research Electronic Data Capture) survey system.

The trial was preregistered at ClinicalTrials.gov (NCT04426318) and through the Open Science Framework [50]. However, the current prediction analyses were not planned a priori and were not included in the preregistered data analysis plan. All code (implemented in the R statistical software [51]) used to reproduce the analyses in the manuscript have been posted on Open Science Framework [52].

The HMP includes contemplative practices designed to build skills supportive of 4 pillars of well-being: awareness, connection, insight, and purpose [36,37]. Participants were encouraged to engage with content from each of the 4 modules for approximately 1 week (ie, 4 weeks total). The content included didactic instruction and guided meditation practices. For the guided practices, participants could select the length of practice from 5 to 30 minutes. The HMP app was used for a mean of 10.9 (SD 9) days during the 4-week trial. For additional trial and sample details, refer to the study by Hirshberg et al [31].

### Ethics Approval

The study procedures were approved by the University of Wisconsin—Madison Institutional Review Board (number 2020-0533).

### Measures

#### Demographic Characteristics

The participants reported their age, gender identity, race and ethnicity, marital status, and income at baseline.

#### Primary Outcome

The prespecified primary outcome in the parent RCT was psychological distress, which was a composite of the computer-adaptive versions of the PROMIS Anxiety and PROMIS Depression measures [35] and the 10-item Perceived Stress Scale [38]. All 3 are widely used measures with established reliability and validity [39,40]. Refer to Multimedia Appendix 1 for details. Consistent with the prespecified data analytic plan, multilevel models estimated changes in distress over the 4-week intervention period. Random slopes (representing individual changes in distress over the intervention period) were calculated for each participant and served as the primary outcome in our machine learning prediction models.

#### Predictors

Several additional self-report questionnaires assessed secondary outcomes and candidate mediators that were theoretically linked to the pillars of well-being trained within the HMP. The 15-item Perseverative Thinking Questionnaire (PTQ) [53] assessed worry and rumination (Cronbach *α*=.95). The 5-item World Health Organization [54] assessed global well-being (*α*=.85). The 8-item Act with Awareness subscale of the Five Facet Mindfulness Questionnaire [55] assessed mindful attention in daily life (*α*=.91). The 5-item National Institutes of Health Toolbox Loneliness Questionnaire [56] assessed perceived social disconnection (*α*=.90). The 12-item Self-Compassion Scale Short Form [57] assessed feelings of kindness toward oneself (*α*=0.86). The 10-item Drexel Defusion Scale [58] assessed the ability to experientially distance from internal experiences (*α*=.84). The 10-item Meaning in Life Questionnaire (MLQ [59]) assessed the presence and search for meaning (Cronbach *α*=.91 and Cronbach *α*=.93, respectively).

### Analytic Strategy

Predictor variables included preintervention distress (composite measure), anxiety (PROMIS), depression (PROMIS), stress (Perceived Stress Scale), repetitive negative thinking (PTQ), the mindfulness facet of acting with awareness (Five Facet Mindfulness Questionnaire), loneliness (National Institutes of Health Toolbox Loneliness), defusion (Drexel Defusion Scale), presence (MLQ), search for meaning (MLQ), self-compassion (Self-Compassion Scale Short Form), well-being (5-item World Health Organization), age, gender, race, marital status, and income.

#### Missing Value Imputation

Missing data were imputed using a random forest–based imputation (MissForest package in R [60]). To avoid contamination between the predictor and outcome scores, which may optimistically bias predictive performance, the outcome variable (slope of change in distress) was excluded from the imputation procedure. The rate of missing data was very low, with no variable missing more than 6 values. Refer to Multimedia Appendix 1 for additional details.

#### Generating Predicted Outcomes

To predict outcomes, 2 prognostic models (using elastic net regularized regression [ENR]; glmnet package in R) were developed: one for participants who received HMP and one for those who received the assessment-only control condition. To minimize overfitting, which can occur with traditional *k*-fold CV, a nested CV procedure was used for each of these prognostic models (ie, incorporating an outer and inner CV loop [41-44]). Refer to Multimedia Appendix 1 for details of the nested CV procedure.

The steps mentioned earlier generated predicted HMP outcomes for HMP participants and predicted control condition outcomes for the control participants. To generate predicted outcomes for the counterfactual condition (ie, the treatment condition one did not receive), an ENR model was developed for one group (ie, full HMP or control sample) and used to predict outcomes for participants in the other group.

#### Evaluation of Recommendations

As a final product of the prediction models mentioned earlier, every participant had 2 predicted outcome scores: one for HMP and one for the control condition. Consistent with previous similar studies [18,19,61], we computed a PAI score by subtracting these 2 predicted outcomes (ie, the predicted slope of change in distress for HMP *minus* control) for each individual. Thus, a negative PAI score indicates that a given participant is predicted to experience greater reductions (ie, a more negative slope) in distress in HMP relative to the assessment-only control condition (and vice versa for positive PAI scores). The PAI can be interpreted as a continuous indicator reflecting the expected magnitude of the advantage of one treatment condition relative to the other (eg, a large negative PAI value indicates that the model predicts a relatively large between-group difference in outcome favoring HMP). We tested whether PAI scores moderated treatment group differences in outcome (ie, slope of change in distress) via a group (ie, intervention condition) × PAI interaction. The latter test allowed us to answer the following question: Are more negative PAI scores (reflecting relatively greater predicted benefit from HMP relative to the control condition) in fact associated with larger *observed* differences in outcomes favoring HMP?

#### Comparison Model

We compared the abovementioned multivariable machine learning (ENR) model with a simple linear regression with baseline repetitive negative thinking (PTQ) scores as the sole predictor (ie, repeating the above steps to generate a PAI score for every participant) implemented via 10-fold CV (repeated 100 times to generate stable estimates). Repetitive negative thinking was selected as a predictor in this comparison model based on prior research, indicating that it predicts response to mindfulness apps [43,45]. Refer to Multimedia Appendix 1 for additional analyses with baseline distress as the sole predictor. Finally, we used the parameter estimates from the final models to demonstrate the translation of the predicted outcomes to *personalized* recommendations for app-based mindfulness training.

All analyses were conducted using R software (version 4.0.2) [62]. The sample size was originally determined for the purpose of the parent trial to detect between-group differences in the primary outcome (change in distress [50]). To estimate whether the current sample size was adequately powered for the analyses proposed in this study, a Monte Carlo simulation approach (InteractionPoweR package in R) was used. Informed by the effect sizes from a prior mindfulness app RCT [45] that tested similar group × PAI interactions, simulations revealed that a sample size of at least 153 was needed for group × PAI interaction tests (with Cronbach *α*=.05; power=80%; refer to Figure S1, including figure note, in Multimedia Appendix 1 for additional power analysis details).

## Results

### Sample Demographics

The majority (523/662, 79%) of the participants reported depression or anxiety symptoms at baseline that were above the clinical cutoff on the PROMIS Depression and PROMIS Anxiety measures (*t* score>55), and more than half of the sample (343/662, 51.8%) reported moderate or greater anxiety or depressive symptoms at baseline (*t* score>60).

The groups did not differ at baseline in terms of the demographic or clinical variables (Table 1). Of those assigned to HMP, 95.6% (329/344) downloaded the app and 78.8% (271/344) used the app for ≥1 day. The mean number of days of use was 10.88 (SD 9.08). The mean number of minutes of practice was 127.93 (SD 130.63).

**Table 1:** Descriptive statistics for Healthy Minds Program and assessment-only control at baseline.

Variable		Healthy Minds Program				Control				*P*^a^ value
		Value, N	Value, n (%)	Value, mean (SD)	Value, N		Value, n (%)	Value, mean (SD)		
Age (years)		344	—	42.47 (11.06)	318		—	42.70 (10.23)	.78	
Gender (female)		344	299 (86.9)	—	318		279 (87.7)	—	.75	
Non-Hispanic White		344	304 (88.4)	—	318		268 (84.3)	—	.13	
Married		344	243 (70.6)	—	318		216 (67.9)	—	.45	
College education		343	308 (89.8)	—	316		281 (88.9)	—	.72	
**Income (US $)**										
	≤50,000	344	56 (16.3)	—	318		55 (17.3)	—	.73	
	50,000-100,000	344	141 (41.0)	—	318		129 (40.6)	—	.91	
	100,000-150,000	344	104 (30.2)	—	318		96 (30.2)	—	.99	
	≥150,000	344	40 (11.6)	—	318		37 (11.6)	—	.99	
**PROMIS^b^**										
	Depression	342	—	55.37 (6.20)	315		—	55.47 (6.43)	.85	
	Anxiety	342	—	59.83 (6.95)	315		—	60.00 (7.11)	.75	
Perceived Stress Scale		342	—	2.89 (0.56)	315		—	2.87 (0.60)	.69	
Distress^c^ (composite)		342	—	0.00 (0.88)	315		—	0.00 (0.91)	.97	
Perseverative Thinking Questionnaire		342	—	29.89 (10.43)	315		—	29.62 (11.29)	.76	
Five Facet Mindfulness Questionnaire—Acting with Awareness Subscale		342	—	24.80 (5.93)	315		—	24.56 (6.12)	.62	
National Institutes of Health Toolbox Loneliness		342	—	2.53 (0.77)	315		—	2.58 (0.77)	.45	
Drexel Defusion Scale		342	—	24.83 (7.89)	315		—	24.50 (8.16)	.60	
**MLQ^d^**										
	Presence	342	—	26.20 (5.44)	315		—	25.81 (5.46)	.36	
	Search for meaning	342	—	21.63 (6.61)	315		—	22.09 (6.79)	.38	
World Health Organization well-being		341	—	12.76 (4.71)	315		—	12.47 (4.33)	.42	
Self-Compassion Scale		342	—	2.98 (0.69)	315		—	2.93 (0.70)	.37	

^a^*P* value from independent samples *t* test comparing groups at baseline.

^b^PROMIS: Patient-Reported Outcomes Information System.

^c^Distress: composite of PROMIS Depression, PROMIS Anxiety, and Perceived Stress Scale.

^d^MLQ: Meaning in Life Questionnaire.

### Outcome Prediction

Higher baseline levels of distress, depression, and stress predicted better outcomes (ie, greater reductions in distress) in HMP (Table 2). The zero-order correlations between outcome and these 3 predictors were *r=*−0.30 (for distress), *r=*−0.30 (depression), and *r=*−0.26 (stress). Predicted HMP outcomes were significantly correlated with observed outcomes for the HMP group (*r*=0.27; *P*<.001; root mean square error [RMSE]=0.10) but not with the control condition outcomes (*r*=0.07; *P*=.21; RMSE=0.12). Conversely, predicted control condition outcomes were significantly correlated with observed outcomes for the control group (*r*=0.19; *P*<.001; RMSE=0.10) but not with HMP outcomes (*r*=0.10; *P*=.06; RMSE=0.12). Higher baseline scores for the following variables predicted better outcomes in the control condition: distress, anxiety, depression, stress, loneliness, defusion, and presence. In addition, lower levels of repetitive negative thinking, higher self-compassion, and being married were associated with better control condition outcomes (Table 2).

**Table 2:** Baseline variables retained in elastic net models predicting outcomes for each condition^a^.

Predictors		Healthy Minds Program model, coefficient	Control model, coefficient
Age (years)		—^b^	—
Gender		—	—
Race		—	—
Marital status		—	−0.006
Income		—	—
**PROMIS^c^**			
	Depression	−0.012	−0.005
	Anxiety	—	−0.007
Perceived Stress Scale		−0.003	−0.006
Distress^d^ (composite)		−0.011	−0.008
Perseverative Thinking Questionnaire		—	0.012
Five Facet Mindfulness Questionnaire—Acting with Awareness Subscale		—	—
National Institutes of Health Toolbox Loneliness		—	−0.002
Drexel Defusion Scale		—	−0.011
**MLQ^e^**			
	Presence	—	−0.008
	Search for meaning	—	—
World Health Organization well-being		—	—
Self-Compassion Scale		—	−0.002

^a^The larger set of baseline predictors retained in the elastic net regularized regression model applied to the control participants relative to the Healthy Minds Program (HMP) group was because the best-fitting model in the former group had a lower *α* value (ie, closer to ridge than lasso regression) relative to the HMP group. Negative parameter estimates indicate that higher scores on the predictor variable are associated with better outcomes (ie, reductions in distress).

^b^Variables that were not retained in the elastic net model.

^c^PROMIS: Patient-Reported Outcomes Information System.

^d^Distress: composite of PROMIS Depression, PROMIS Anxiety, and Perceived Stress Scale.

^e^MLQ: Meaning in Life Questionnaire.

### Meditation App Recommendations

The mean PAI score was −0.07 (SD 0.03; range −0.17 to 0.03), indicating that the model predicted greater average symptom improvement for the HMP meditation app than for the assessment-only control condition. The model recommended HMP (PAI<0) for all participants except 5 (657/662, 99.2%).

### Evaluation of Recommendations

**Figure 1:**
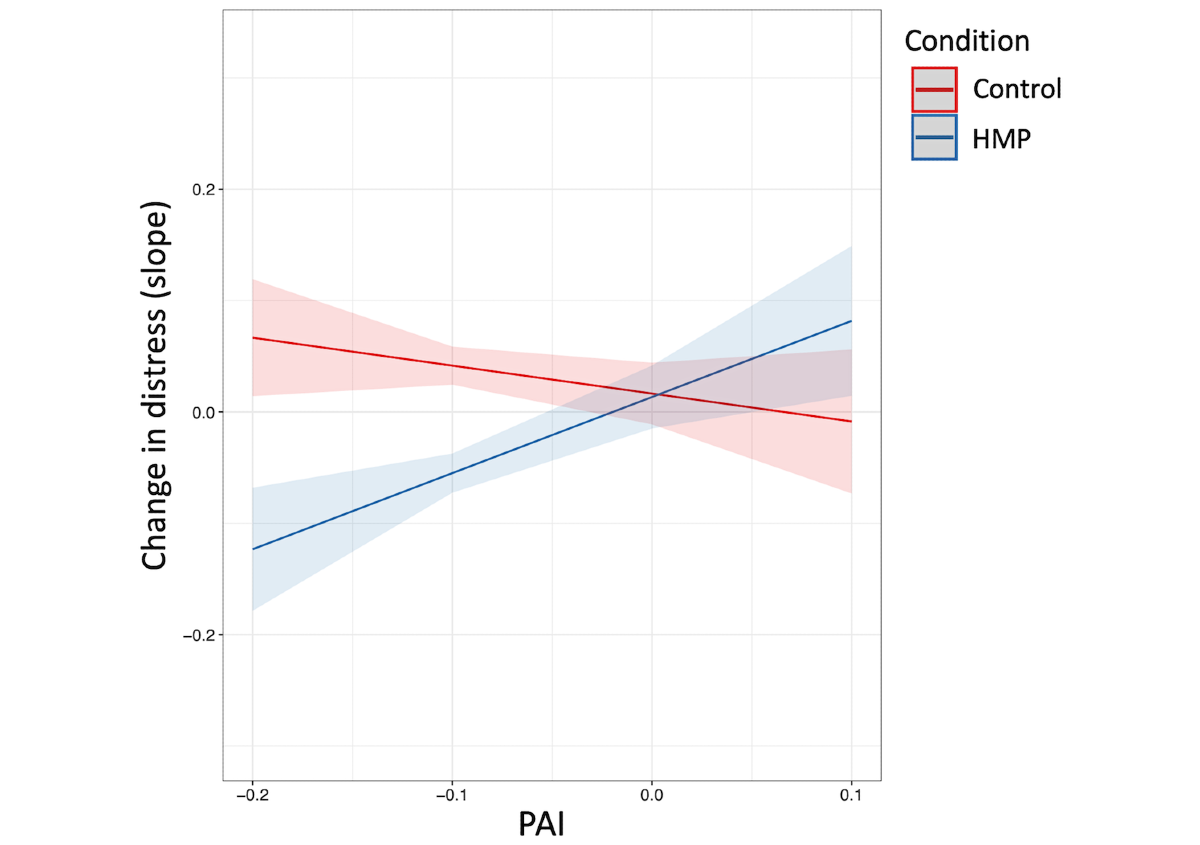
Group × Personalized Advantage Index (PAI) interaction. As PAI scores decrease (ie, reflecting relatively stronger recommendations for the Healthy Minds Program [HMP] app) group differences in observed outcome increase, favoring HMP.

A significant group × PAI interaction emerged in predicting outcome (*t*_658_=3.30; *P*=.001; adjusted *r*^2^=0.10), indicating that PAI scores moderated group differences in outcome. As displayed in Figure 1, as PAI scores decrease (ie, reflecting relatively stronger HMP recommendations), group differences in observed outcome increase, favoring HMP.

### Comparison Model

In the linear regression comparison model applied to the HMP group, higher levels of repetitive negative thinking were significantly associated with a *greater* reduction in distress from the mindfulness app (B*=*−0.02; *t*_342_=−3.37; *P*<.001). The correlation between predicted HMP outcomes and observed outcomes was *r*=0.16 (*P*=.003; RMSE=0.10) for participants who received HMP and *r*=−0.14 (*P*=.02; RMSE=0.12) for the control group. In contrast to the pattern of findings for the HMP group, the linear regression model applied to the control sample revealed that higher levels of repetitive negative thinking were significantly associated with *poorer* outcomes than in the control condition (B*=*0.01; *t*_316_=2.44; *P*=.02).

The correlation between predicted control condition outcomes and observed outcomes was *r*=0.11 (*P*=.049; RMSE=0.11) for the control group and *r*=−0.18 (*P*<.001; RMSE=0.12) for the HMP group.

**Figure 2:**
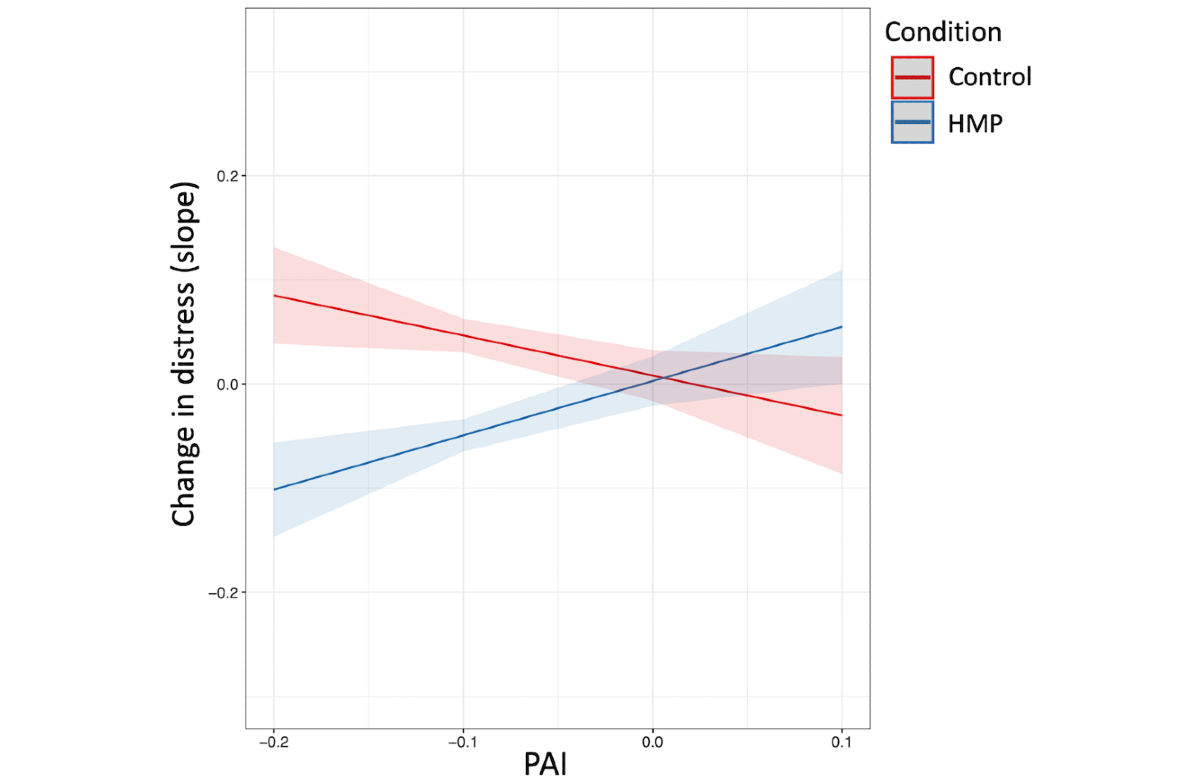
Group × Personalized Advantage Index (PAI) interaction for the comparison model (ie, linear regression with baseline repetitive negative thinking [PTQ] scores as the sole predictor). As PAI scores decrease (ie, reflecting relatively stronger recommendations for the Healthy Minds Program [HMP] app) group differences in observed outcome increase, favoring HMP.

A significant group × PAI interaction emerged in predicting changes in distress (*t*_658_=3.81; *P*<.001; adjusted *r*^2^=0.11), indicating that PAI scores moderated group differences in outcomes (Figure 2). Specifically, as PAI scores decreased (reflecting increasing repetitive negative thinking scores), group differences favoring the HMP condition also increased. Given the association between repetitive negative thinking and depressive symptoms [46,47], we also conducted additional sensitivity analyses controlling for baseline levels of depressive symptoms (as well as considering the number of days the app was used), which yielded the same pattern of findings (Multimedia Appendix 1). In summary, these results indicate that a simple linear regression including repetitive negative thinking as the sole predictor yields equivalent performance relative to a more complex multivariable ENR model (ie, adjusted *r*^2^=0.11 vs *r*^2^=0.10, respectively, for the group × PAI interaction).

### Translating a Predictive Model to Personalized Meditation App Recommendations

To demonstrate the translation of a predictive model to personalized recommendations, we used the parameter estimates from the above regression models to estimate predicted changes in distress in HMP versus the assessment-only condition for a new individual based on their preintervention repetitive negative thinking score. Given that the simpler regression model performed similarly to the more complex multivariable ENR models, we used the former model for this demonstration.

**Figure 3:**
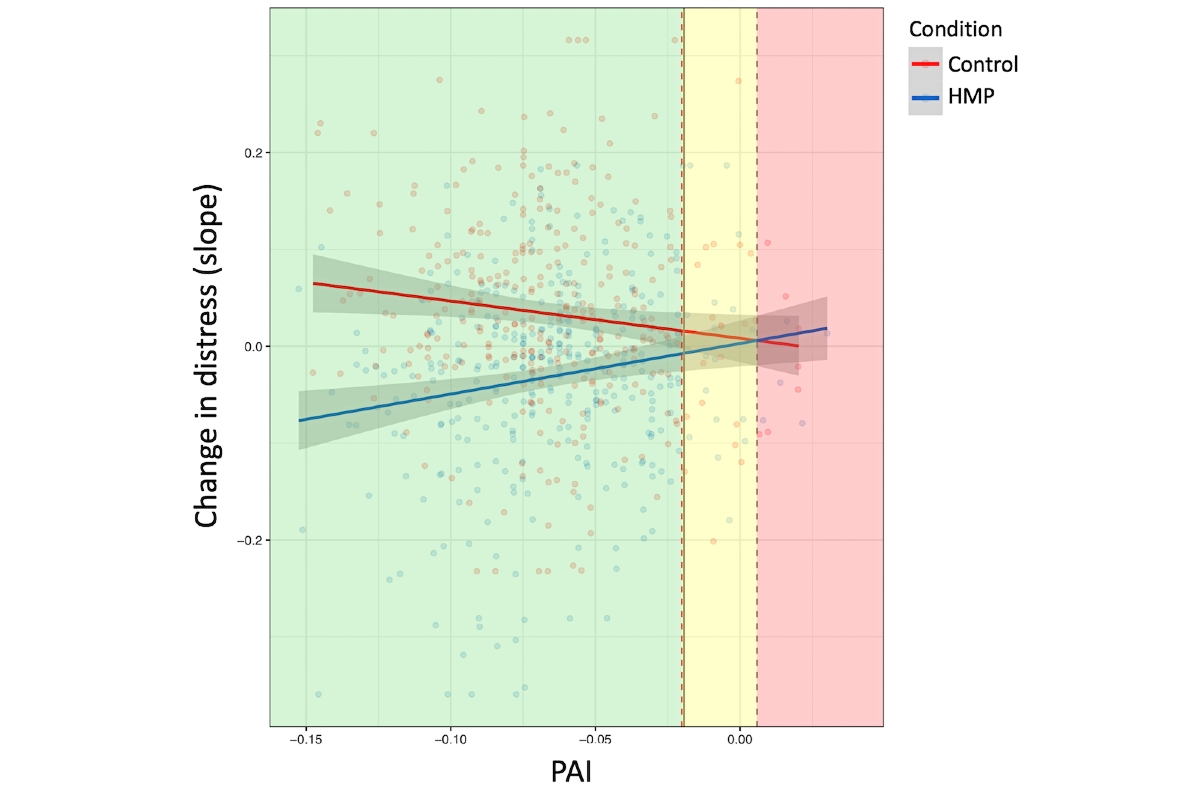
Plot of the relationship between Personalized Advantage Index (PAI) scores and outcome for each condition to inform personalized recommendations. The dashed vertical gray line indicates the point at which the 2 regression lines intersect (left margin of a bootstrapped 95% CI is shown with a dashed vertical red line). The solid vertical gray line (adjacent to the red line) is derived from the Johnson-Neyman technique and represents the value of the moderator (PAI) at which between-group differences in outcome become statistically significant. Refer to the detailed description in text, with an example for personalized Healthy Minds Program [HMP] recommendation.

First, as shown in Figure 3, we plotted the relationship between PAI scores and outcomes for HMP (blue line) and the assessment-only control condition (red line). The dashed vertical gray line represents the point at which the 2 regression lines intersect. An individual with a PAI score to the left of this line was predicted to have a better outcome in HMP relative to the assessment-only control condition (and vice versa for individuals with PAI scores to the right of this line). The area to the left of this line is colored yellow, reflecting a “cautious recommendation” for app-based meditation training. Second, we computed a 95% CI via bootstrap resampling (Boot package in R) [63]. Specifically, we drew 1000 samples with replacement and recomputed the 2 regression lines and their intersection points in each of these samples. The dashed vertical red line represents the left margin of the 95% CI for this intersection point. In other words, if an individual’s PAI score falls to the left of this line, our confidence in the predicted benefit of HMP relative to the assessment-only condition increases. Third, we also implemented the Johnson-Neyman technique [64] (Interactions package in R) to probe the group × PAI interaction and to estimate the value of the moderator (PAI) at which group differences in outcomes become statistically significant. This occurred at PAI<−0.02 (solid vertical gray line in Figure 3, immediately adjacent to the dashed red line). If a participant’s PAI score falls to the left of both the 95% CI (dashed red line) and the Johnson-Neyman threshold (solid gray line), the plot area is colored green to reflect a more confident recommendation to use HMP.

To illustrate with a concrete example, an individual with a repetitive negative thinking (PTQ) score of 1 SD above the mean (ie, 41) would have a PAI score of −0.10 (within the “green zone” of Figure 3) and a predicted slope of change in distress of −0.049 (ie, expected reduction in distress) in HMP and 0.047 (ie, expected increase in distress) in the assessment-only condition over 4 weeks. Assuming that this individual had a preintervention level of distress at the 50th percentile, they would be predicted to be at the 41st percentile (relative to preintervention distress scores) following the 4-week mindfulness app course and at the 58th percentile if they only completed symptom assessments (ie, control condition). In summary, based on a brief assessment of perseverative negative thinking, our algorithm can provide individual users with useful information regarding their expected benefit before they decide to enroll in a multiweek course of app-based meditation training.

## Discussion

### Principal Findings

An increasing number of individuals are turning to meditation apps to alleviate their emotional distress. Meditation apps represent the most commonly used mental health apps for depression and anxiety [30]. Despite their growing popularity, little is known about the benefits of these apps. In this study, we developed an algorithm to predict the benefit that an individual would be expected to experience from a smartphone-based meditation intervention (HMP) relative to an assessment-only control condition. We found evidence that a data-driven model can successfully predict differential response to a meditation app versus an assessment-only control condition using self-reported baseline demographic and clinical characteristics. Specifically, PAI scores significantly moderated group differences in outcomes. Individuals with more negative PAI scores, reflecting relatively stronger meditation app (ie, HMP) recommendations, had better outcomes if randomly assigned to the meditation app relative to the control condition. As expected, given overall group (ie, HMP > control) differences in outcome [31], the models typically predicted greater benefits from HMP versus the control condition. However, the predicted benefits of HMP were not always large, and in some cases, the PAI model predicted either relatively small between-group differences in outcome (“yellow zone” in Figure 3) or even better outcomes in the control condition (“red zone”). The former cases could be interpreted as instances in which the costs of engaging in a multiweek meditation app course (eg, time investment, delay in engaging with other, more helpful interventions) may not be worth the potential benefits.

Critically, a comparison linear regression model that only included information about baseline levels of repetitive negative thinking performed comparably well to a multivariable machine learning model (in contrast, refer to the studies by Webb et al [65] and Buckman et al [66]). Repetitive negative thinking moderated the outcome of app-based meditation training relative to the assessment-only control condition. Importantly, these findings reveal that higher repetitive negative thinking is not simply a general “prognostic” indicator of one’s likelihood of experiencing reductions in distress (eg, due to regression to the mean or the passage of time). In other words, greater repetitive negative thinking did not predict greater reductions in distress in *both* the meditation app and control conditions. Instead, and similar to prior research focused on a different mindfulness app and sample (adolescents with elevated rumination) [43,45], individuals with higher baseline levels of repetitive negative thinking derived greater relative benefit from a meditation app. One question is whether these findings are specific to repetitive negative thinking or instead may be driven by correlated clinical characteristics, in particular, depressive symptoms or distress. Sensitivity analyses revealed that repetitive negative thinking significantly moderated group differences in outcomes even when controlling for depressive symptom severity or distress (Multimedia Appendix 1). In summary, these findings indicate that a brief self-report assessment of repetitive negative thinking can inform which individuals are most likely to benefit from app-based meditation training.

As illustrated in Figure 3, our predictive model can be readily applied for personalized meditation app recommendations for new individuals. First, the model provides a binary prediction of whether an individual is expected to experience greater reductions in distress from the meditation app relative to symptom assessment only (ie, based on whether PAI scores fall to the left or right of the intersection point [vertical dashed gray line]). Second, the model provides an estimate of the *magnitude* of the expected difference in outcomes between the meditation app and the control condition. Finally, the model also distinguishes between the strengths of recommendations to use the meditation app, demarcated by the green (confident recommendation) and yellow (cautious recommendations) zones of the figure (with boundaries defined by a bootstrapped CI and Johnson-Neyman interval). Collectively, this information can be used to provide individuals with objective metrics about expected outcomes to inform their decision on whether to enroll in a meditation app course. Such information could readily be implemented within mHealth interventions such as the HMP. Participants could first complete a brief self-report assessment of repetitive negative thinking and receive feedback on their predicted outcomes before deciding to use the app.

Although potentially useful in terms of encouraging the optimal use of users’ time and attention, informing some individuals that engagement with a meditation app may not be beneficial to them is unlikely to be embraced by many intervention developers. However, these models can be readily extended to instances in which one or more mHealth interventions are being compared. Given the thousands of available mental health apps [25], which should be compared? One approach is to focus on the most popular (eg, most frequently downloaded) mental health apps, which include mindfulness, journaling, CBT, and mood tracking apps [29,30]. For example, future studies could develop algorithms for predicting response to various popular mental health apps, which differ substantially in intervention focus (eg, meditation apps vs CBT-based apps vs mood tracking) [29,67], or even compare a mental health app with conventional (in-person) psychotherapy or pharmacotherapy. Such studies could determine, for example, whether we can predict which individuals with depressive symptoms require conventional, face-to-face CBT (or an antidepressant prescription) versus those who would experience symptom remission from a brief app-based meditation or CBT course. In addition, future studies could compare different versions of a single app. For example, individuals may differ in the extent to which they benefit from different types of meditation (eg, cultivating focused attention on breath, open monitoring, or loving-kindness meditations) or different lengths or frequencies of guided meditation sessions.

In addition to informing consumer choice, the ability to predict who is most likely to benefit from a particular intervention could inform health care policy and decision-making. In contrast to a stepped care model in which treatment intensity is escalated based on the response to interventions, predictive models could be used to initially assign patients to the treatment expected to yield the best outcomes for that individual based on their baseline characteristics (ie, stratified care) [68]. In theory, the latter approach could minimize the delay in receiving an effective intervention.

Another important avenue for future research is to test the extent to which these findings can be generalized to other meditation apps (eg, Headspace and Calm). In many ways, HMP is similar to other meditation apps. It includes training in mindfulness and connection (eg, loving-kindness, compassion) practices that are also available in popular mindfulness apps such as Headspace and Calm. One difference is that HMP includes practices designed specifically to cultivate a healthy sense of self (Insight module) as well as purposes and meaning in life (Purpose module). The inclusion of these practices is derived from a neuroscience-based model of well-being on which HMP is based [36]. Thus, it is more accurate to view HMP as a meditation app that intentionally moves beyond mindfulness to place equal emphasis on other domains of well-being and contemplative practices designed to support these additional domains. Ultimately, additional research is needed to test whether the pattern of findings presented in this study generalize to other meditation and mindfulness apps.

Finally, given the lack of prior research predicting mental health app outcomes, further research is needed to test the impact of presenting predicted mindfulness app prognosis on patient outcomes. For example, before using a mindfulness app, patients could be randomly assigned to receive their predicted outcomes or not receive this information. Several relevant outcomes could be examined, including (1) between-group differences in symptom change, (2) the extent to which receiving these predictions influences expectancies of therapeutic benefit, (3) the relationship between expectancies and app outcome, and (4) the extent to which individuals use the algorithm-recommended intervention or disregard the recommendation.

### Limitations

This study has several important limitations. First, although basing models exclusively on self-reported data is attractive from an implementation perspective, we may have excluded other patient characteristics that provide important additional predictive information to inform optimal treatment recommendations (eg, biomarkers and cognitive tasks) [12]. In addition, repetitive negative thinking, which emerged as a predictor of differential response, may be more validly assessed via methods other than conventional retrospective self-report questionnaires (eg, repeated, daily ecological momentary assessment [43,69]). Other relevant variables (eg, app use data, motivational variables, and involvement in other activities linked to better mental health) could be assessed in future studies. Second, our results emerged within a specific sample (school district employees), which did not have adequate representation of males, Black, Indigenous, and people of color, or individuals with low income. The sample is representative of Wisconsin in terms of race (83% of Wisconsinites are White) but includes a higher proportion of females. However, the gender difference in our sample is not surprising, given that females are more likely than males to (1) be employed as teachers [70] and (2) experience and seek treatment for depressive and anxiety symptoms [71]. Third, we were unable to conduct external validation by evaluating model prediction performance in an entirely new sample (eg, from another RCT). Fourth, as is common in digital therapies [48], a sizable subset of participants used the app for relatively few days. However, the results remained significant when restricting our analyses to subsets of participants who used the app for a longer period (Multimedia Appendix 1). Fifth, we did not include an active comparison condition. Our assessment-only control condition was not designed to control for placebo-related processes [72]. The methods demonstrated here may ultimately be most relevant in helping patients and clinicians decide between competing interventions that are intended to be therapeutic.

### Conclusions and Future Directions

This study demonstrated the potential utility of data-driven approaches for informing personalized meditation app recommendations. A natural extension of this study is to conduct a prospective test of our algorithm using a doubly randomized design. For example, participants could be randomized to either (1) random treatment assignment (ie, treatment A or treatment B) or (2) be assigned to their algorithm-indicated treatment. To the extent that patient outcomes are significantly (and clinically meaningfully) better in the latter condition, the results would support the clinical benefits of algorithm-informed treatment recommendations (for a recent example of a similar design testing predictive matching of patients to therapists, refer to the study by Constantino et al [73]). In addition to comparing treatment packages, this design could be readily used to evaluate other customizable elements of HMP or other mHealth interventions. This may include assignment to receive various components or ordering of components within HMP, assignment to HMP or an alternative commonly used mHealth intervention (eg, CBT, behavioral activation, journaling, or mood tracking apps), or assignment to varying treatment intensities (eg, meditation practice frequency).

Other potentially fruitful future directions include evaluating a broader set of patient characteristics previously shown or hypothesized to predict the likelihood of responding to different interventions [5]. In addition, prediction models could be developed using data drawn from large naturalistic data sets evaluating mHealth interventions, as has been done for in-person psychotherapy and pharmacotherapy [65,74-76]. In addition to testing the utility of these models in “real-world” settings, naturalistic settings often provide large data sets relative to RCTs and thus can increase statistical power [28]. Ultimately, these approaches may gradually help supplement our reliance on trial and error for treatment selection with empirically supported, data-driven algorithms to objectively communicate expected benefits to individuals, allowing them to make well-informed decisions about which interventions are best for their needs.

## Appendix

Multimedia Appendix 1 Supplement.

Multimedia Appendix 2 CONSORT e-HEALTH checklist.

## Abbreviations

CBT: cognitive behavioral therapy
CONSORT: Consolidated Standards of Reporting Trials
CV: cross-validation
HMP: Healthy Minds Program
mHealth: mobile health
MLQ: Meaning in Life Questionnaire
PAI: Personalized Advantage Index
PROMIS: Patient-Reported Outcomes Information System
PTQ: Perseverative Thinking Questionnaire
RCT: randomized controlled trial
REDCap: Research Electronic Data Capture
RMSE: root mean square error
