# Supplementary material for: Personalized Prediction of Response to Smartphone-Delivered Meditation Training: Randomized Controlled Trial
Source: J Med Internet Res. 2022 Nov 8;24(11):e41566. doi: 10.2196/41566 (PMC9682449; doi:10.2196/41566)

**­Supplementary Online Content**

**Personalized prediction of response to smartphone-delivered meditation training**

**Supplemental Methods**

**Healthy Minds Program (HMP)**

The HMP includes contemplative practices designed to build skills supportive of four pillars of well-being: awareness, connection, insight, and purpose [1,2]. Briefly, awareness includes skills in attention regulation and meta-awareness; connection involves intra- and interpersonal relational skills including gratitude, kindness, and compassion; insight is structured around an accurate understanding of how beliefs regarding identity and self shape experience; and purpose involves clarifying values and applying them in daily life activities. Participants were encouraged to engage with content from each of the four modules for approximately one week (i.e., 4 weeks total). Content included didactic instruction as well as guided meditation practices. For the guided practices, participants could select the length of practice from 5 to 30 minutes. The HMP app was used for a mean of 10.9 days (SD = 9) over the 4-week trial. Within digital therapies, attrition and low uptake is a common challenge [3]. In the present study, 73% of the sample used the app for 2 or more days (69% for 3 or more days, 67% for 4 or more days). For additional trial and sample details, see [4].

**Primary Outcome**

The prespecified primary outcome in the parent RCT was psychological distress which was a composite of the computer-adaptive versions of the PROMIS Anxiety and PROMIS Depression measures [5] and the 10-item Perceived Stress Scale (PSS) [6]. All three are widely used measures with established reliability and validity [7,8]. Internal consistency of the PSS was adequate in the current sample (a = .86). A composite was calculated by averaging across standardized (i.e., z-scored) versions of the three measures (standardized using baseline means and standard deviation [SD])(Given that we used the computer adaptive PROMIS, Cronbach’s alpha cannot be computed for this measure).

**Post-Imputation Procedures**

Following the multiple imputation, and prior to conducting the prediction analysis, continuous variables were z-standardized (mean = 0, SD = 1) and categorical variables (i.e., marital status, gender and race) dummy coded (-0.5 and 0.5). Given the small percentage of non-White participants in this sample (86% non-Hispanic White), race was coded as White or non-White. To reduce the influence of outliers, we winsorized extreme values (Winsorize function in the DescTools R package) by setting values below the 1^st^ percentile and above the 99^th^ percentile to the 1^st^ percentile and 99^th^ percentile values, respectively [9].

**Generating predicted outcomes**

To predict outcomes, two prognostic models were developed, one for participants who received HMP and one for those who received the assessment-only control condition. To minimize overfitting which can occur with traditional *k*-fold cross-validation (CV), a nested CV procedure was used for each of these prognostic models (i.e., incorporating an outer and inner CV loop [10–13]). For the nested CV, we first split the data into 10 folds (10 training/test sets), representing the outer CV loop. For *each* of the latter outer training sets, the above set of predictor variables were submitted to 10-fold CV (i.e., the “inner” CV loop) elastic net regularized regression (ENR; glmnet package) to generate predictions of outcome (repeated 100 times to generate stable estimates). Specifically, each of the outer training samples were split into 10 equal-sized samples and predicted outcomes for each of the held out 1/10 of the training sample were generated from an ENR model developed in the other 9/10ths of the data. ENR’s alpha (which controls the balance between ridge regression [alpha = 0] and LASSO [alpha = 1]) and lambda (which controls the extent to which predictor coefficients are shrunk) parameters were tuned via the CARET package’s tuneLength parameter which was set to 20 resulting in 400 combinations of alpha and lambda values. The combination of alpha and lambda that minimized root mean squared error (RMSE; estimated with the inner CV) was selected, and a final model was fit on the entire outer training set and used to predict outcomes for the participants in the outer test set. Importantly, the nested CV procedure ensures that predicted HMP and control condition outcomes for all participants were generated from ENR models that were constructed without the use of their own data.

**Supplemental Results**

**Sample Demographics.** Consistent with the demographics of Wisconsin school district employees, the sample was predominantly female (86.9%) and non-Hispanic White (86.1%). Most were married (69.5%) and had completed a college degree (89.2%). The most common income bracket was US$50,000-US$100,000 (40.9%), followed by US$100,000-US$150,000 (30.4%).

**Sensitivity Analyses.** Several alternative baseline comparison models were run for the purpose of sensitivity analyses. First, in the main text we report a significant Group x PAI interaction in predicting symptom change. We re-ran this analysis controlling for baseline distress which yielded the same pattern of findings (*t*(657) = 3.07, *P =* .003; adjusted *r*^2^ = .14).

Second, given the association between repetitive negative thinking and depressive symptoms [14,15], we also conducted a sensitivity analysis controlling for baseline levels of depressive symptoms, which yielded the same pattern of findings (Group x PAI interaction, (*t*(657) = 3.67, *P <* .001; adjusted *r*^2^ = .14). Third, we reran the comparison PAI models substituting baseline distress scores (i.e., pre-intervention scores on the outcome measure) as the sole predictor in the model for baseline repetitive negative thinking scores, which yielded the same significant interactions, though slightly attenuated (Group x PAI interaction, (*t*(658) = 2.20, *P =* .028; adjusted *r*^2^ = .13). Fourth, we tested whether baseline repetitive negative thinking scores moderated group differences in outcome after controlling for a baseline depressive symptom severity x group interaction. The baseline repetitive negative thinking x group interaction remained significant (*t*(656) = -2.61, *P =* .009), whereas the baseline depression x group interaction was not significant (*t*(656) = -1.28, *P =* .200). Similarly, the baseline repetitive negative thinking x group interaction remained significant (*t*(656) = -2.19, *P =* .029) when controlling for a baseline distress x group interaction (*t*(656) = -0.99, *P =* .320). Finally, consistent with elevated attrition and low uptake from digital therapies,[3] 73% of the sample used the app for 2 or more days (69% for 3 or more days, 67% for 4 or more days). Consistent with the primary outcome report [4], we included all subjects in our intent-to-treat analyses. However, we re-ran the primary group x PAI interaction on a dataset restricted to those whose used the app 2 or more days. The interaction remained significant (*t*(559) = 3.20, *P =* .001). Similarly, results remained significant when restricting our results to subsamples using the app for 3 or more days (*t*(544) = 3.11, *P =* .002), for at least one day per week (i.e., 4 or more days total)(*t*(539) = 3.14, *P =* .002) or at least once daily (i.e., 28 or more times)(*t*(316) = 3.59, *P <* .001).

**Supplementary References**

1. Dahl CJ, Wilson-Mendenhall CD, Davidson RJ. The plasticity of well-being: A training-based framework for the cultivation of human flourishing. Proc Natl Acad Sci 2020 Dec 22;117(51):32197–32206.

2. Goldberg SB, Imhoff-Smith T, Bolt DM, Wilson-Mendenhall CD, Dahl CJ, Davidson RJ, Rosenkranz MA. Testing the Efficacy of a Multicomponent, Self-Guided, Smartphone-Based Meditation App: Three-Armed Randomized Controlled Trial. JMIR Ment Health 2020 Nov 27;7(11):e23825. [doi: 10.2196/23825]

3. Torous J, Nicholas J, Larsen ME, Firth J, Christensen H. Clinical review of user engagement with mental health smartphone apps: evidence, theory and improvements. Evid Based Ment Health Royal College of Psychiatrists; 2018;21(3):116–119.

4. Hirshberg MJ, Frye C, Dahl CJ, Riordan K, Vack N, Sachs J, Goldman RI, Davidson RJ, Goldberg S. A Randomized Controlled Trial of a Smartphone-Based Well-Being Training in Public School System Employees During the COVID-19 Pandemic [Internet]. PsyArXiv; 2021 [cited 2021 Jun 26]. [doi: 10.31234/osf.io/hrvmu]

5. Pilkonis PA, Choi SW, Reise SP, Stover AM, Riley WT, Cella D, Group PC. Item banks for measuring emotional distress from the Patient-Reported Outcomes Measurement Information System (PROMIS®): depression, anxiety, and anger. Assessment Sage Publications Sage CA: Los Angeles, CA; 2011;18(3):263–283.

6. Cohen S. Perceived stress in a probability sample of the United States. Sage Publications, Inc; 1988;

7. Roberti JW, Harrington LN, Storch EA. Further psychometric support for the 10‐item version of the perceived stress scale. J Coll Couns Wiley Online Library; 2006;9(2):135–147.

8. Schalet BD, Pilkonis PA, Yu L, Dodds N, Johnston KL, Yount S, Riley W, Cella D. Clinical validity of PROMIS depression, anxiety, and anger across diverse clinical samples. J Clin Epidemiol Elsevier; 2016;73:119–127.

9. Steyerberg EW. Clinical Prediction Models: A Practical Approach to Development, Validation, and Updating. 2nd ed. 2019 edition. Cham, Switzerland: Springer; 2019. ISBN:978-3-030-16398-3

10. Moons KG, Altman DG, Reitsma JB, Ioannidis JP, Macaskill P, Steyerberg EW, Vickers AJ, Ransohoff DF, Collins GS. Transparent Reporting of a multivariable prediction model for Individual Prognosis or Diagnosis (TRIPOD): explanation and elaboration. Ann Intern Med American College of Physicians; 2015;162(1):W1–W73.

11. Varoquaux G, Raamana PR, Engemann DA, Hoyos-Idrobo A, Schwartz Y, Thirion B. Assessing and tuning brain decoders: Cross-validation, caveats, and guidelines. NeuroImage 2017 Jan 15;145:166–179. [doi: 10.1016/j.neuroimage.2016.10.038]

12. Webb CA, Swords CM, Murray LM, Hilt LM. App-based Mindfulness Training for Adolescent Rumination: Predictors of Immediate and Cumulative Benefit. Mindfulness 2021 Jan 15;12:2498–2509. [doi: https://doi.org/10.1007/s12671-021-01719-0]

13. Wetherill RR, Rao H, Hager N, Wang J, Franklin TR, Fan Y. Classifying and Characterizing Nicotine Use Disorder with High Accuracy Using Machine Learning and Resting-State fMRI. Addict Biol 2019 Jul;24(4):811–821. PMID:29949234

14. Watkins ER, Nolen-Hoeksema S. A habit-goal framework of depressive rumination. J Abnorm Psychol 2014 Feb;123(1):24–34. PMID:24661156

15. Watkins ER, Roberts H. Reflecting on rumination: Consequences, causes, mechanisms and treatment of rumination. Behav Res Ther 2020 Apr 1;127:103573. [doi: 10.1016/j.brat.2020.103573]

16. Webb CA, Swords CM, Lawrence H, Hilt LM. Which adolescents are well-suited to app-based mindfulness training? A randomized clinical trial and data-driven approach for personalized recommendations [Internet]. J Consult Clin Psychol. 2022 [cited 2021 Jul 21]. [doi: 10.31234/osf.io/7gbjd]

**Supplemental Figures**

**Figure S1.** Results of Monte Carlo simulations (1,000 iterations; implement via InteractionPowerR package in R). We inputted four *r*-type effect sizes (based on [16]) representing the correlation between the interaction term (Group x PAI) and outcome (*r* = .22), the correlation between Group and PAI scores (*r* = - .12) and their respective main effects (*r* = - .21; *r* = .15). The yellow curve plots power at varying sample sizes (80% power at n = 153). Two additional sets of simulations were conducted assuming an interaction effect size 75% (magenta; 80% power at n = 278) and 50% (blue; 80% power at n = 599) the magnitude of the above effect size. The black horizontal line represents 80% power.

**Figure S2.** CONSORT Flow Diagram. HMP = Healthy Minds Program; WLC = waitlist control (i.e., assessment only control); T1 = baseline, T2 = week 1, T3 = week 2, T4 = week 3, T5 = post-test, T6 = 3-month follow-up. Measures administered at these timepoints included clinical scales (depression, anxiety, stress, and loneliness), as well as measures of mindfulness, cognitive defusion, and meaning in life. For additional clinical trial and participant details (including CONSORT Checklist) see [4], https://psyarxiv.com/hrvmu/


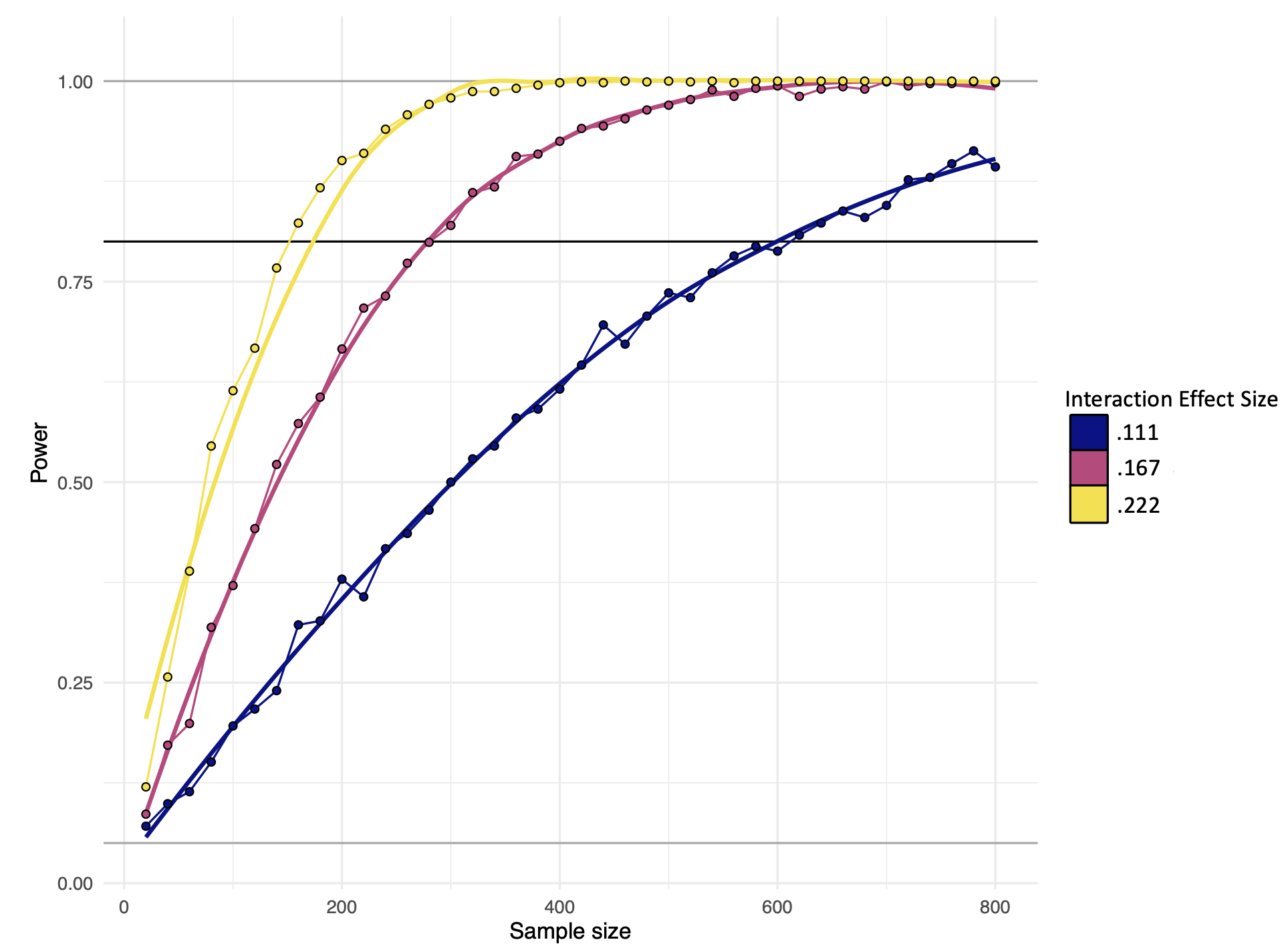

Supplement: Multimedia Appendix 1 [file jmir_v24i11e41566_app1.docx]
